# Tumor Necrosis Factor Receptor 1 Expression Is Upregulated in Dendritic Cells in Patients with Chronic HCV Who Respond to Therapy

**DOI:** 10.1155/2010/429243

**Published:** 2010-08-03

**Authors:** Raul Cubillas, Katherine Kintner, Frances Phillips, Nitin J. Karandikar, Dwain L. Thiele, Geri R. Brown

**Affiliations:** ^1^Division of Digestive and Liver Diseases, Department of Internal Medicine, Southwestern Medical Center at Dallas, University of Texas at Dallas, Dallas, TX 75390-9151, USA; ^2^Dallas Veterans Affairs Medical Center, 4500 S. Lancaster Road, Dallas, Texas 75216, USA

## Abstract

The present studies assessed the level of tumor necrosis factor receptor (TNFR) expression in peripheral blood mononuclear cells (PBMCs) subsets from patients with chronic HCV undergoing interferon *α*/ribavirin-based therapy (Ifn/R). *Methods*. TNFR family member mRNA expression was determined using quantitative real-time PCR assays (RTPCRs) in PBMC from 39 HCV+ patients and 21 control HCV− patients. Further subset analysis of HCV + patients (untreated (U), sustained virological responders (SVR), and nonresponders (NR)/relapsers (Rel)) PBMC was performed via staining with anti-CD123, anti-CD33, anti-TNFR1 or via RTPCR for TNFR1 mRNA. *Results*. A similar level of TNFR1 mRNA in PBMC from untreated HCV+ genotype 1 patients and controls was noted. TNFR1 and TNFR2 mRNA levels in PBMC from HCV+ patients with SVR were statistically different than levels in HCV(−) patients. A significant difference was noted between the peak values of TNFR1 of the CD123+ PBMC isolated from SVR and the NR/Rel. *Conclusion*. Upregulation of TNFR1 expression, occurring in a specific subset of CD123+ dendritic cells, appeared in HCV+ patients with SVR.

## 1. Introduction

Unlike infections by other human hepatitis viruses, hepatitis C virus (HCV) infection of healthy immunocompetent adults is persistent or prolonged in the majority of cases, unlike hepatitis B, where it persist in the minority of cases. HCV appears to have evolved strategies to evade the human immune responses. Investigators have suggested that viral interactions with tumor necrosis factor/tumor necrosis factor receptor (TNF/TNFR) family members may be important in immune evasion. Investigators have reported that HCV core protein binds to the cytoplasmic domain of TNFR1, specifically the “death domain,” which may hinder or promote cell death during HCV infection [1]. In addition, the HCV core protein binds to another TNFR family member, lymphotoxin *β* receptor's (LT*β*R) intracellular domain, which is involved in signal transduction [2]. Investigators have also demonstrated that the HCV core protein potentiates NF*6*B activation initiated by TNF*α*/TNFR and LT*α*_1_ *β*_2_/LT*β*R interactions, which, in turn may contribute to the chronically activated, persistent state of HCV-infected cells [3, 4]. Interference with signaling through members of the TNF family of receptors may affect the chronicity of HCV via affecting either cell death and/or activation of NF*κ*B [1, 3, 4].

Of interest, other investigators have examined the relationship between HCV and the soluble TNF receptors (*s*TNFR) in the blood. These sTNFR levels appear to peak 9 hours after the first IFN*α* administration correlating with IFN*α* serum levels [5]. Other investigators have noted higher levels of TNF and *s*TNFRs in HCV+ patients than in HCV (−) controls prior to treatment and postulated that high levels of *s*TNFR might modify host responses but found no correlation between levels and response to therapy [6].

TNF receptors are present on the surface of a number of cell subsets and play a role in a variety of functions. For example, TNF/TNFR interactions are imperative for the optimal proliferation and effector functions of CD8 +  T cells [7, 8], whose antiviral effects are essential for the clearance of noncytopathic viruses, such as HCV. In addition, LT*β*R-LIGHT/LT*α*_1_ *β*_2_ interactions have been implicated in both optimal growth and effector functions as well as costimulation of CD8+ T cells [9]. Similarly, TNFR is expressed on macrophages, peripheral blood monocytes, and antigen presenting cells (APCs), cells known to be important in viral infections. Recently, HCV negative strand RNA has been noted in the macrophages of 67% of sustained virological responders to interferon-based therapies [10]. Others have noted the importance of monocytes and dendritic cells in the clearance of HCV [11–14]. The role of TNFR in HCV viral clearance may involve any of these cell subsets.

The largely chronic nature of HCV infection has been attributed to an attenuated antiviral T-cell response. It has been postulated that APC's may become dysfunctional in some way during HCV infection contributing to this attenuation. Specifically, large deficits in IFN*α* production in plasmacytoid dendritic cells (PDCs) in HCV-infected patients have been reported in [15]. In the same study HCV-infected PDC displayed distinct immunophenotypic features including an increased ability to stimulate a mixed lymphocyte response (MLR) but lower HLA-DR and CD86 expression. This profile suggested that HCV-infected PDC were at an immature stage of differentiation [15]. 

The present studies were designed to quantify the mRNA and protein levels of the TNF receptor family members, including TNFR1 and TNFR2, in PBMCs during IFN*α*-based therapy of HCV+ patients. The results of these studies reveal increased peak levels of TNFR1 mRNA levels in responders to IFN-based therapies. Furthermore, by flow cytometric studies and western blot analysis, upregulation of TNFR1 protein expression was noted in specific PBMC subsets. The increase in TNFR1 expression was specifically isolated in PDC (CD11− and CD123^high^) of HCV-infected patients who responded to IFN*α* based therapies compared to control patients and nonresponders.

## 2. Methods

### 2.1. Patients

 Veteran patients >18 years of age with chronic HCV were recruited to IFN*α* based treatment trials conducted at the Dallas Veterans Affairs Medical Center from February 2002 through July 2005. Thirty-seven patients with genotype 1 consented per institutional review board (IRB) guidelines for blood draws at one or more time points, including 0, 4, 8, 12, 24, and 48 weeks of therapy, where the standard therapy (48 weeks of weight-based dosing of 1.5 *μ*g/kg of pegylated interferon *α*-2b and 13.5 mg/kg of (ribavirin) for genotype 1 patients was utilized. Thirteen patients were sustained virological responders (SVR, HCV RNA negative 24 weeks after end of therapy), 20 patients were nonresponders (NR, persistently HCV RNA positive during therapy), and 4 patients were relapsers (Rel, HCV RNA negative during therapy but HCV RNA positive after end of therapy). None of the patients analyzed had received interferon prior to the initial blood draw. Similarly, 10 HCV(−) patients and 11 patients with alcoholic liver disease (ALD) or nonalcoholic fatty liver disease (NAFLD) consented per IRB guidelines for a blood draw. 

In a second set of patients, samples from 13 genotype 1 HCV+ patients (5 SVR, 6 NR/Rel and 2 HCV+ prior to therapy) and 2 HCV(−) control patients obtained during therapy at <12, 16–24, or 25–44 weeks were collected for a specialized cell subset analysis.

### 2.2. RNA Isolation and Real-Time PCR Primers

 RNA was isolated from PBMC's at 0, 4 8, 12, 24, and 48 weeks of interferon/ribavirin (Ifn/R) treatment trials from 39 HCV+ men, 10 age- and sex- matched HCV(−) controls, and 11 age- and sex- matched controls with either alcoholic liver disease (ALD) or nonalcoholic fatty liver disease (NAFLD). Initially, 40–60 cc of blood was obtained from HCV+ patients and controls. PBMCs were then separated by density gradient centrifugation and then stored in TRIzol reagent to stabilize the RNA. RNA was then extracted, precipitated, and subjected to DNase treatment, and then the treated RNA underwent reverse transcription. 

In order to assess whether there were differences in any specific cytokine receptors or TNF receptor family members, 101 cDNA samples were utilized in a quantitative real time PCR-based assay assessing the relative quantities of cyclophilin, NGFR, CD30, TNFR1, TNFR2, LT*β*R, IL-2R*α*, CD27, FAS, and CD3. RNA from PDC (CD11− and CD123^high^) and myeloid dendritic cells (MDC) (CD11+ and CD123^dim ^) from a control, SVR, and NR were isolated in an identical fashion and utilized in a quantitative PCR assay for TNFR1. 

The primers utilized were obtained as follows. The NCBI database was first interrogated to find the sequence of the cytokine receptor or other receptors of interest and then entered into the Primer Express software program in order to generate a primer which was then optimized according to the parameters of the real time PCR (Table 1). The threshold cycle or **C**_**t**_ value, the lowest cycle in which fluorescence was detected during the exponential phase, was calculated where the exponential phase of the reaction began.

### 2.3. Standardization and Comparison of Samples

 The C_t_ values were standardized using RNA isolated from cell lines known to express high levels of the target genes. In order to normalize across experiments, these values were compared to the same standard for all experiments, that is RNA from cell lines expressing high levels of TNFR or LT*β*R (PHA activated lymphocytes and THP-1, resp.). The resulting cycle threshold values were normalized to endogenous cyclophillin values and a calibrator value for each experiment. Raw cycle threshold values for the no template control within each experiment were significantly different than the experimental values for cyclophillin, CD3*ε*, NGFR, CD30, TNFR1, TNFR2, LT*β*R, FAS, IL-2R*α*, and CD27, verifying the presence of cDNA for these receptors.

The comparison between control HCV(−) patients and SVR, NR, and Rel were expressed as fold elevation above control. The control population is defined as both HCV(−) patients with ALD or NAFLD (*n* = 9) and HCV(−) control subjects (*n* = 10).

### 2.4. Western Blot Analysis

Whole PBMC's from one SVR with a high level of TNFR1 mRNA by real time PCR were analyzed by Western blot in order to determine protein levels of TNFR1. Whole PBMC's were freeze-thawed twice, suspended in PBS, and assayed for protein, and 20 *μ*g from one SVR and one control as well as 200 ng of recombinant human soluble TNF receptor 1 (R&D Systems, Minneapolis/St. Paul, MN) were prepared for sodium dodecyl sulfate-polyacrylamide gel electrophoresis (SDS-PAGE) in the presence of 2-mercaptoethanol, resolved on a 10% acrylamide gel, and transferred to a nitrocellulose membrane. After blocking in 5% milk in TBS (0.1 mol/L NaCl and 0.4 mmol/L Tris, pH 7.4), containing 0.05% sodium azide, the blot was exposed to human soluble TNF receptor 1 polyclonal detection antibody (R&D Systems). Bound antibody was allowed to react with alkaline phosphatase-conjugated goat antimouse IgG (Sigma Chemical Co., St. Louis, MO) [15].

### 2.5. Flow Cytometry

At 44 weeks of therapy, whole PBMCs from a SVR and an NR were isolated from 40–60 cc whole blood by density gradient separation. In another experiment, PBMCs from an SVR at 48 weeks, NR at 24 weeks, and control patient were isolated as previously described for subset analysis. The PBMC's from all patients were suspended in staining buffer (0.5 L HBSS, 0.5 L PBS, 2% BSA, 0.02% sodium azide). Cells were labeled with the FITC-labeled anti-TNFR1 Ab (FAB225F, R&D Systems) or control FITC IgG_1_6 (555748l, BD Pharmingen). The samples designated for cell subset analysis were then stained with CD33 (555748l, BD Pharmingen), CD14 (555748l, BD Pharmingen), CD11 (555748l, BD Pharmingen), CD123 (555748l, BD Pharmingen), or relative isotype controls. After mAb staining and washing, all samples were fixed in PBS containing 1% paraformaldehyde at room temperature for 10 minutes and stored at 4°C until flow cytometric analysis as previously described in [16, 17]. Flow cytometric analysis was performed on an FACscan (Becton Dickinson) and used to determine the mean fluorescence intensity (MFI) of TNFR1 on all labeled cells. TNFR1 MFI ratios (TNFR1 MFI/isotype control MFI) were determined. 

RNA from FAC sorted plamacytoid dendritic cells (PDCs) (CD11− and CD123^high^) and myeloid dendritic cells (MDCs) (CD11+ and CD123^dim ^  from a control, SVRs and NR were utilized in a real time PCR assay for TNFR1.

### 2.6. Statistical Analysis

**Figure 1:**
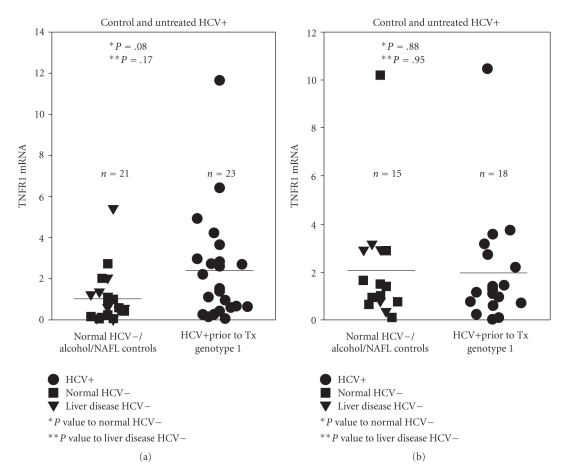
TNFR1 and TNFR2 mRNA levels were not different between HCV(−)/ALD/NAFLD control patients and genotype 1 HCV+ patients prior to treatment. TNFR1 mRNA levels of PBMC's isolated from 10 HCV(−) controls, 11 ALD/NAFLD controls, and 23 HCV+ patients prior to therapy were ascertained by real time PCR (Panel A). TNFR2 mRNA levels of PBMC's isolated from 10 HCV(−) controls, 5 ALD/NAFLD controls, and 18 HCV+ patients prior to therapy were ascertained by real time PCR (Panel B). Statistical differences were assessed by *T*- test.

**Figure 2:**
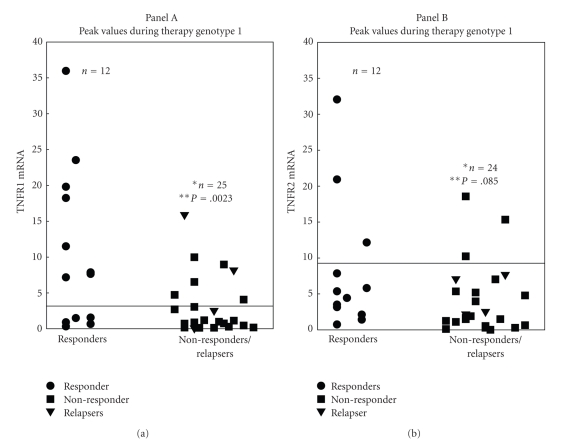
Peak TNFR1 mRNA values were higher in responder patients than nonresponder patients. TNFR1 mRNA levels of genotype 1 HCV+ patients PBMC's were ascertained by real time PCR. Twelve responders, 25 NR/Rel and 12 responders, 24 NR/Rel were assessed for TNFR1 and TNFR2 mRNA levels, respectively (Panel A and B). Line indicates 2 SD above mean control levels. Levels did not vary between sample triplicates. Statistical differences were assessed by *T* test.

 Descriptive data were reported as a mean for all standard deviation (SD) and median and range in Figures 1 and 2. All results were analyzed with an intention to treat analysis. Characteristics of treatment groups were compared using student's *t*-test. All results were analyzed using an exact Fisher test. A “*P*” value of less than .05 was considered to be significant.

## 3. Results

### 3.1. PBMC Tumor Necrosis Factor Receptor (TNFR) mRNA Levels from Responders to IFN Therapy Increased while Other TNFR Family Members mRNA Levels Remained Stable

 RNA from PBMC of 23 HCV+ patients was isolated prior to the commencement of IFN*α* based therapy. Similarly, RNA from PBMC's of 10 HCV(−) controls and 11 patients with ALD or NAFLD were also isolated. During the course of 48 weeks of IFN*α* based therapy, 80 RNA samples were isolated from PBMC's from 13 HCV+ responder [(SVR and end of treatment responders (ETR)] patients and from 24 NR patients. Quantitative real time PCR levels of TNFR1, TNFR2, LT*β*R, FASR, CD30, CD27, and NGF, as well as CD3*ε* and IL-2R*α* mRNA were assessed in available samples at 8, 12, 24, and/or 48 weeks of therapy (Table 2). In order to normalize across experiments, these values were normalized to the same standard for all experiments, that is, RNA from cell lines expressing high levels of TNFR (PHA-activated lymphocytes).

As noted, TNFR1 mRNA levels of PBMC from HCV+ responder patients were significantly higher than levels from control age- and sex- matched HCV(−) control patients during the course of the IFN*α* based therapy (*n* = 26, *P* = .033), while TNFR1 mRNA levels of PBMC's from HCV NR patients were not significantly different from controls (*n* = 38, *P* = .20). Similarly, during the course of IFN*α* based therapy, TNFR2 mRNA levels of PBMC from HCV+ responder patients were significantly higher than levels in the control age- and sex- matched HCV(−) patients (*n* = 22, *P* = .045) while no difference was noted relative to levels in HCV NR and controls (*n* = 33, *P* = .41). No other significant increases were observed in the other TNFR family members or T-cell-associated mRNA (CD3*ε* or IL-2R*α*) levels of PBMC isolated from responders or NR (Table 2). In summary, PBMC's mRNA levels of TNFR1 and TNFR2 from responders to IFN*α* based therapy were statistically different than levels in control HCV(−) age- and sex- matched patients.

### 3.2. TNFR1 and TNFR2 mRNA Levels from HCV+ Patients Prior to Treatment and Control Patients Were Similar

In light of differences between levels of TNFR1 and TNFR2 mRNA in PBMC of responders and nonresponders during the therapy, further analysis of the TNFR1 and TNFR2 levels was performed. RNA was isolated from PBMCs of 23 HCV+ untreated patients, 10 control HCV(−) patients, and 11 patients with ALD or NAFLD. TNFR1 and TNFR2 mRNA levels were similar in both HCV infected and control groups [1.02 ± 1.25 (*n* = 21) versus 2.53 for all 2.71 (*n* = 23) and 1.95 for all 2.42 (*n* = 15) versus 2.07 for all 2.45 (*n* = 18)] (Figure 1, panels A and B). Quantitative real time PCR revealed no difference between initial levels of TNFR1 mRNA in the HCV+ genotype 1 patients and control non-HCV+ patients (*P* = .08) nor between HCV+ genotype 1 patients and patients with non-HCV liver disease (*P* = .17). Similar results between initial TNFR1 RNA levels in control (HCV negative and non-HCV liver disease) and initial levels in HCV+ patients were noted [median 0.59 (Range: 0.02–5.42)] versus [median 1.52 (Range: 0.06–11.65)] (Figure 1, panel A). TNFR2 mRNA levels were not significantly different between HCV+ patients prior to therapy and either HCV(−) control patients or HCV(−) patients with liver disease (*P* = .88 and  .95), (Figure 1, panel B).

### 3.3. Peak TNFR1 mRNA Levels Were Significantly Different between SVR and Nonresponders

 In order to determine whether the elevation of TNFR1 or TNFR2 mRNA was above control levels at any point during IFN*α* based therapy (4, 8, 12, 24 and 48 weeks), 12 genotype 1 HCV+ SVR and 25 genotype 1 HCV+ patients NR/Rel were evaluated. TNFR1 and TNFR2 mRNA elevations were significantly more frequent in SVR versus NR/Rel.

The peak TNFR1 mRNA levels in PBMC of genotype 1 HCV+ patients at 4, 8, 12, 24, or 48 weeks were greater than 2 SD (2.63 fold elevation) above mean TNFR1 mRNA levels from HCV(−) controls in 7/12 (58%) sustained virological responders (SVR), but in only 5/21 (28%) genotype 1 NR and 2/4 (50%) genotype 1 Rel. Furthermore, the peak TNFR1 mRNA levels from responders were significantly higher than the peak TNFR1 mRNA levels in NR and Rel (*P* = .0023) [SVR: Median 7.37 (Range: 0.29–35.92) versus NR/Rel: Median 1.17 (Range: 0.08–15.91)] (Figure 2(a)). 

The peak TNFR2 mRNA levels expressed in PBMC from genotype 1 HCV+ patients at 4, 8, 12, 24 or 48 weeks were greater than 2 SD (8.6 fold elevation) above mean control TNFR2 mRNA levels from HCV(−) patients in 3/12 (25%) SVR versus 3/20 (15%) genotype 1 NR and 0/4 (0%) genotype 1 Rel. The peak TNFR2 mRNA levels from SVR were not significantly higher than the peak TNFR2 mRNA levels from NR and Rel (*P* = .085) (Figure 2(b)). Similar results of peak TNFR levels in responders and nonresponders were noted [median/range 6.1 (0.71–32.01) versus 2.98 (0.01–18.57) in Figure 2(b)]. Therefore, unlike peak PBMC TNFR1 mRNA levels, peak PBMC TNFR2 mRNA levels during IFN for all therapy were not significantly different between SVR and nonresponders.

### 3.4. TNFR1 Expression by HCV+ SVR Patient's PBMCs Was Demonstrated by Western Blot Analysis

**Figure 3:**
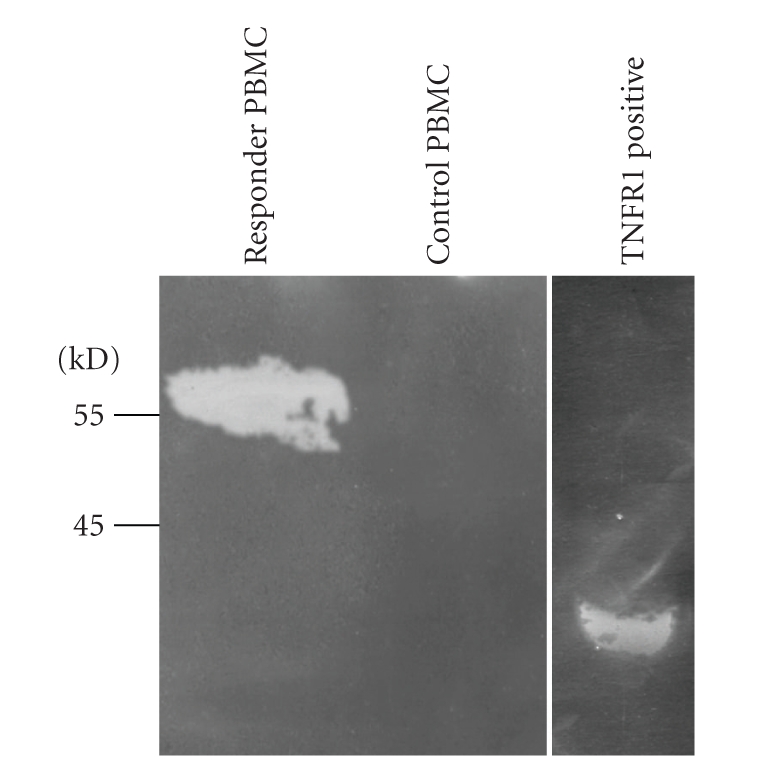
TNFR1 protein expression was observed in PBMC's from HCV+ responder patient. Whole PBMC's from a responding HCV+ patient and a control HCV(−) patient were lysed and assayed for protein content. Protein (20 *μ*g) was loaded for responder (lane 1) and HCV(−) patient (lane 2) and 0.2 *μ*g TNFR1 chimeric protein positive control (lane 3) was loaded and subjected to SDS-PAGE.

In order to assess whether TNFR1 mRNA levels directly correlated with differential expression of TNFR1 protein in a SVR or control PBMCs, whole PBMC from a HCV+ SVR and a control HCV(−) patient were lysed, assayed for protein content, and 20 *μ*g of protein per sample was loaded on a SDS PAGE gel. Detection of the protein of −55 kDa molecular weight by anti-TNFR1 was observed in the PBMC of an HCV+ responder patient but was not observed in the HCV(−) control PBMC lysate (Figure 3).

### 3.5. Increased TNFR1 Membrane Expression by Specific Peripheral Blood Monocyte Subsets from SVR but Not NR Was Observed

**Figure 4:**
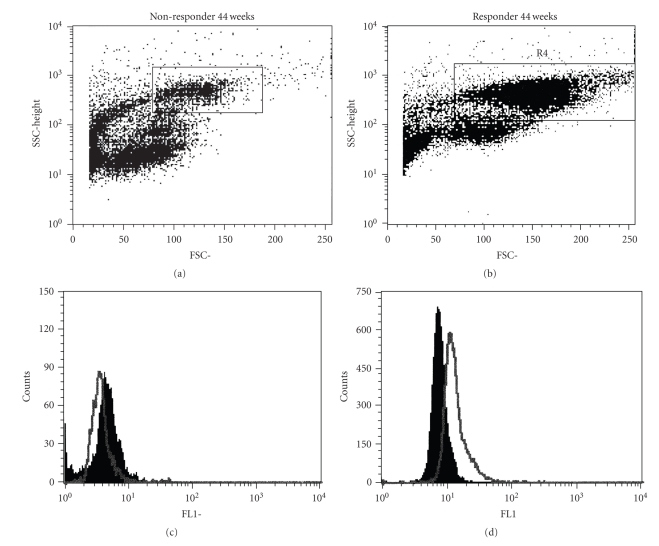
Flow cytometry revealed an increased frequency of PBMC's that expressed TNFR1 among responders. Whole PBMCs were stained with FITC TNFR1 as described in the methods. Dot plots are noted on (a) and (b) with regions analyzed on each dot plot. (c) and (d) demonstrate the responder and the nonresponder histograms of TNFR1 in the specific region outlined in (a) and (b).

The surface expression of TNFR1 by PBMC in HCV+ patients undergoing IFN/R has not been reported. However, soluble TNFR1 has been described to be elevated in patients with chronic HCV [6]. Because of the differences noted in the TNFR1 mRNA levels between NR and the SVR, flow cytometric analysis of TNFR1 expression by PBMC from a SVR and a NR was assessed. Whole PBMCs from NR and SVR were isolated and stained with an FITC-conjugated anti-TNFR1 Ab as described in the methods. The PBMC of the HCV+ SVR patient displayed 52% TNFR1 positive PBMC with mean fluorescence intensity (MFI) of 80. The non-responding patient displayed 0.0% TNFR1 positive PBMC's, with MFI of 0. As demonstrated by the flow cytometric figure, the increase of TNFR1 levels was noted in cells with light scatter characteristics of peripheral blood monocytes or dendritic cells (Figure 4).

In order to further delineate which PBMC subset had elevated levels of TNFR1 expression, TNFR1 MFI ratios (TNFR1 MFI/isotype control MFI) were compared between SVR, NR/Rel, and controls. HCV+ patients prior to therapy and uninfected controls were noted to have similar TNFR1 MFI ratios in both the CD33+ (1.2 ± 0.01 (*n* = 2) versus 1.56 ± 0.37 (*n* = 2), resp. *P* = .19) and CD123+ PBMC (1.14 ± 0.56 (*n* = 2) versus 1.40 ± 0.09, (*n* = 2) resp., *P* = .57). No difference was observed between the TNFR1 MFI ratios from both CD33+ and CD123+ PBMC of the uninfected controls and the NR (*P* = .4 and *P* = 1.0, resp.). 

In order to further assess the specific cell subset affected by IFN*α*, 24 PBMC samples were stained with CD123, CD33, and TNFR-1 (16 SVR and 8 NR/Rel). Sixteen samples were obtained from 5 SVR from weeks 62–48, and 8 samples were taken from NR/Rel from weeks 5–81. A trend toward a lower TNFR1 expression in CD123+ dendritic cells subset was noted in the NR/Rel group (1.25 ± 0.45 versus 1.59 ± 0.42, *P* = .052). Of interest, a significant difference was noted between the peak value of TNFR MFI ratio (highest TNFR1 MFI ratio observed during therapy) in CD123+ PBMC isolated from SVR and NR/Rel (Table 3).

**Figure 5:**
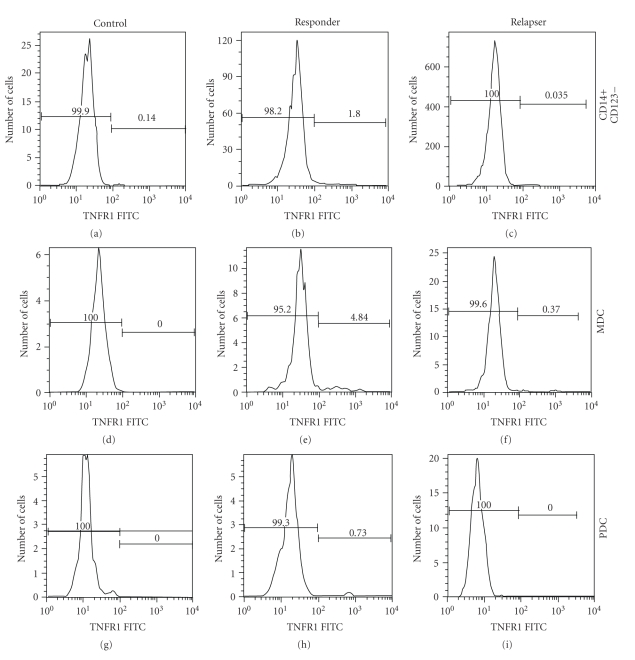
TNFR1 membrane expression is higher in dendritic cell subset in SVR compared to NR. Whole PBMCs from control HCV−, SVR and NR were stained with PE-labeled CD123, FITC-labeled CD11, Texas Red-labeled CD14, and/or relative isotype controls as previously described in the methods. CD11+, CD123^dim ^  , CD11−, CD123^high^, and CD14+, CD11+ cell subsets were identified by 2-color analysis. Significant differences were noted in the expression of TNFR1 in the histogram from selected cell subset from a control HCV− (column a), SVR (column b), and NR (column c).

Further cell subset identification was performed utilizing an analysis by flow sort using three cell markers to identify the two cell subsets of dendritic cells: myeloid (MDC) and plasmacytoid dendritic cells (PDCs). In the first panel, the control patient without chronic liver disease had virtually no TNFR1 expression, in all 3 subsets, CD14+CD123+, MDC (CD11+ and CD123^dim ^), and PDC (CD11− and CD123^high^) (Figure 5(a)). However, in the second panel, after 48 weeks of Ifn/R therapy, expression of TNFR1 in MDC and PDC in the SVR was higher compared to the control (Figure 5(b)) In the non responder group the expression of TNFR1 was similar to controls, low in all three subsets (Figure 5(c)). Hence, upregulation of TNFR1 membrane expression in the dendritic cell subset isolated from the SVR was noted.

### 3.6. TNFR1 mRNA Levels Increased during IFN*α* Based Therapy and Was Isolated to the Plasmacytoid Dendritic Cells

 In order to determine when TNFR1 mRNA levels increased during IFN for all based therapy, 13 genotype 1 SVR and 12 genotype 1 NR or Rel were evaluated. In twenty-five genotype 1 HCV+ patients enrolled in IFN/R therapy with PBMC mRNA samples collected during the 48 weeks of therapy, patients with SVR (*n* = 10) were more likely to have increases in TNFR1 mRNA levels above mean control levels than patients with NR/Rel (*n* = 3) versus 3/12 (25%) NR/Rel. Importantly, the peak levels in the NR/Rel were lower than in the SVR (Tables 4(a) and 4(b)). Of interest, 4/7 (56%) patients with elevated TNFR1 mRNA values during therapy remained above mean control levels weeks after interferon therapy discontinuation.

**Figure 6:**
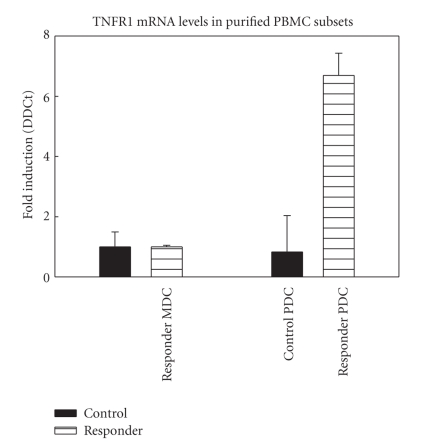
The increase in TNFR1 mRNA levels was isolated to the plasmacytoid dendritic cells. RNA from FAC sorted plamacytoid dendritic cells (PDC) (CD11− and CD123^high^) and myeloid dendritic cells (MDC) (CD11+ and CD123^dim ^) from a control, SVR, and NR were used in a real time PCR assay for TNFR1. Statistical differences were assessed by *T* test.

RNA from FAC sorted plasmacytoid dendritic cells (PDCs) (CD11− and CD123^high^) and myeloid dendritic cells (MDCs) (CD11+ and CD123^dim ^) from a control, SVR, and NR at 28 weeks of therapy were utilized in a real time PCR assay for TNFR1. TNFR1 mRNA levels in PDC isolated from a responder were 6.7 fold higher than the level of the control PDC. No difference in TNFR1 mRNA levels was noted in the control compared to responders MDC, non responders MDC, or non responders PDC. TNFR 1 RNA level is higher in the PDC isolated from patients with SVR than patients with NR at 28 weeks (Figure 6).

## 4. Discussion

This study is the first paper detailing the levels of TNFR family member expression in PBMC samples from responders and nonresponders to IFN therapy for chronic HCV over a 12-month period. Both TNFR1 and TNFR2 mRNA levels in PBMC isolated during therapy were higher in responders than control HCV(−) patients. Notably, the peak values of PBMC TNFR1 mRNA during therapy were higher in sustained virological responders than nonresponders or relapsers. Furthermore, PBMC expression of other members of the TNFR family remained stable during the course of the therapy. Importantly, the TNFR1 mRNA upregulation occurred in a subset of PBMC, with cell surface markers consistent with the dendritic cells.

Though other investigators have noted an increase in soluble TNFR1 (sTNFR1) and soluble TNFR2 (sTNFR2) in the serum of chronic HCV patients (18–21), this study is the first to note that the level of PBMC TNFR mRNA rises over the course of IFN based therapy in responders. Furthermore, others have noted that sTNFR2 appears to be significantly correlated with the severity of the disease and fibrosis [5]. As noted in our previous publications, 50%–60% of patients treated at the Dallas VA have stage 3 fibrosis [18], and in part enhanced TNFR1 expression at baseline may be associated with fibrosis scores. However, there have been no significant differences in fibrosis between responders and nonresponders to therapy in our patient population [18].

Though the upregulation of TNFR1 may be associated with prolonged exposure to IFN, patients with multiple sclerosis (MS) treated with long term type 1 IFN (interferon beta) had stable PBMC TNFR 1 mRNA levels (data not shown). Other mechanisms besides prolonged exposure to type 1 IFN, involved in TNFR1 upregulation may include clearance of HCV virus from specific cell types, allowing further signaling from TNFR1 to NF*6*B, inducing TNFR1 upregulation [4]. Other mechanisms may relate to improved antigen presentation by APC's in specific patient populations after IFN exposure. Finally, specific patient populations may have different genetic polymorphisms of TNFR1, which allow for enhanced viral clearance and upregulation of the receptor's mRNA, accounting for differences in response to IFN based therapy. 

Though there are limitations in the study in that less than half of the patients with SVR had both initial and long term followup, there is a suggestion that TNFR1 RNA levels may increase in patients that respond and that this increase appears be related to an upregulation of TNFR1 on a dendritic cell subset. Though this represents only a portion of the patients, the authors are suggesting that the findings in this study bear reporting.

The finding that dendritic cell subset appears to be the site of TNFR1 upregulation may provide insight into the cause, these cells have been implicated as the cell among PBMC that contains HCV negative RNA strand. Though only a portion of the patients had extensive flow cytometric analysis, the results suggest that the dendritic cell is at least one of the cells, where upregulation of TNFR1 occurs. While recent reports suggest that PBMC's from up to 67% of SVR patients still express residual HCV RNA, these levels are likely greatly reduced from pretherapy levels and relief of HCV-mediated expression of TNFR family signaling may explain rebound enhanced expression of TNFR1. Furthermore, these APC's have also been implicated as being critical for viral clearance. Our study supports that within these cells, a cell surface receptor, TNFR1, is disparately upregulated in patients responding to IFN based therapy.

Initially, TNFR1 expression was noted to be upregulated in CD123+ dendritic cells in patients with sustained virological response. The data supports that at least in some patients the increase in TNFR1 mRNA is predominantly found in the plasmacytoid dendritic cells, an avid antigen presenting cell. Importantly, upregulation of TNFR1 surface expression was noted in one SVR patient's myeloid dendritic cell, suggesting that both types of dendritic cells may be important in HCV viral clearance. 

In summary, TNFR1 mRNA and protein levels are upregulated in dendritic cells. While the mechanism of this upregulation has yet to be elucidated, this cell subset may be involved in antigen presentation of viral proteins, and TNFR1 levels may correlate with recovery of effective APC function.

## Figures and Tables

**Table 1:** Real-Time PCR Primers.

Target	Primer Sequence
Cyclophillin	Fw 5′- TGCCATCGCCAAGGAGTAG -3′
	Rv 5′- TGCACAGACGGTCACTCAAA - 3′
TNFR1	Fw 5′- CGCTACCAACGGTGGAAGTC - 3′
	Rv 5′ -CAAGCTCCCCCTCTTTTTCAG-3′
LT*β*R	Fw 5′- CGGGCCCCTCTAAAGGATT -3′
	Rv 5′- GTGAAGTGTGGAACCCCAAAG -3′
TNFR2	Fw 5′- CAAGCCAGCTCCACAATGG -3′
	Rv 5′- TGACCGAAAGGCACATTCCT -3′
NGFR	Fw 5′- CCGTGGAGATGGGATGCTT -3′
	Rv 5′- TTTCCACGAACCCCAAACC -3′
CD30R	Fw 5′- GCTTTACTCTGGACCATAGGAAACA -3′
	Rv 5′- CTCCTTAGCGTGAAATGTGAAAAA -3′
IL2R*α*	Fw 5′- CAGAAGTCATGAAGCCCAAGTG -3′
	Rv 5′- GGCAAGCACAACGGATGTCT -3′
CD27	Fw 5′- TCGGCACTGTAACTCTGGTCTTC -3′
	Rv 5′- CGACAGGCACACTCAGCATT -3′
FAS	Fw 5′- CTTTTCGTGAGCTCGTCTCTGA -3′
	Rv 5′- CTCCCCAGAAGCGTCTTTGA -3′
CD3	Fw 5′- CATCCCAAAGTATTCCATCTACTTTTC -3′
	Rv 5′- CCCAGTCCATCCCCAGAGA -3′

**Table 2:** TNFR1 Family Members' mRNA Levels.

	ControlHCV neg		Responders*		NR/Rel
		Prior to Tx	On Tx	Prior to Tx	On Tx
TNFR1	0.83 ± 0.9 (*n* = 10) 1.2 ± 1.53 (*n* = 11)**	1.94 for all 2.43 (*n* = 7)	7.09 for all 8.82 (*n* = 26) (*P* = .033)**	2.83 for all 2.87 (*n* = 14)	2.31 for all 3.53 (*n* = 38) (*P* = .20)***
TNFR2	2.10 ± 2.93 (*n* = 10) 2.03 ± 1.34 (*n* = 5)**	1.36 for all 1.22 (*n* = 7)	5.93 ± 7.61 (*n* = 22) (*P* = .045)**	2.30 for all 2.67 (*n* = 12)	2.57 for all 4.60 (*n* = 33) (*P* = .41)**
LT*β*R	1.67 ± 1.01 (*n* = 10)2.17 ± 2.66 (*n* = 10)**	1.21 for all 1.24 (*n* = 7)	1.49 ± 1.64 (*n* = 22) (*P* = .75)**	1.35 for all 1.35 (*n* = 12)	2.47 for all 5.41 (*n* = 34) (*P* = .66)**
FASR	0.38 for all 0.54 (*n* = 3)	0.001 (*n* = 1)	0.14 for all 0.40 (*n* = 18) (*P* = .35)**	0.01 for all 0.01 (*n* = 6)	0.03 for all 0.07 (*n* = 21) (*P* = .003)**
CD30	0.37 for all 0.41 (*n* = 6)	0.175 for all 0.06 (*n* = 2)	0.61 for all 1.22 (*n* = 21) (*P* = .65)**	0.06 for all 0.07 (*n* = 5)	0.61 for all 1.51 (*n* = 20) (*P* = .71)**
CD27	0.86 for all 0.49 (*n* = 6)	0.77 for all 0.37 (*n* = 2)	1.08 for all 0.65 (*n* = 21) (*P* = .45)**	1.10 for all 0.62 (*n* = 5)	0.71 for all 0.62 (*n* = 20) (*P* = .58)**
NGF	0.75 for all 1.22 (*n* = 6)	0.085 for all 0.007 (*n* = 2)	1.85 for all 4.80 (*n* = 22) (*P* = .59)**	0.11 for all 0.19 (*n* = 6)	1.37 for all 2.86 (*n* = 18) (*P* = .62)**
IL-2R for all	0.22 for all 0.39 (*n* = 6)	0.175 for all 0.18 (*n* = 2)	0.45 for all 0.63 (*n* = 21) (*P* = .41)**	0.10 for all 0.22 (*n* = 6)	0.05 for all 0.10 (*n* = 19) (*P* = .09)**
CD3	1.43 for all 0.71 (*n* = 3)	1.3 (*n* = 1)	4.71 for all 12.62 (*n* = 17) (*P* = .67)**	1.67 for all 1.07 (*n* = 5)	6.93 for all 21.06 (*n* = 18) (*P* = .66)**

*Include End of treatment responders (ETR) and SVR.

**Mean value for control liver disease patients, HCV negative.

***P-value compared to control (HCV negative, age and sexed matched).

**Table 3:** 

Peak Values	SVR	NR/Rel	*P* value
TNFR1 MFI Ratio	*N* = 5	*N* = 6	
CD33+	2.2 ± 0.56	1.54 ± 0.57	.06
CD123+	2.06 ± 0.59	1.21 ± 0.43	.01

**Table:** (a)

	week 0	week 4–12	14–24 wks	25–48 wks
Patient 1	0.96	7.83		
Patient 2	1.39	7.13	1.97	0.71
Patient 3	4.23		7.61	3.86
Patient 4			8.44	23.48
Patient 5	0.15		0.63	
Patient 6	6.42		19.77	11.73
Patient 7			0.87	0.82
Patient 8			0.29	1.45
Patient 9			0.15	11.46
Patient 10			1.24	1.52
Patient 11	0.43		35.93	8.04
Patient 12	0.06			18.20
Patient 13		0.29	0.22	

**Table:** (b)

	week 0	week 4–12	14–24	25–48
Patient 1	1.53	4.73		
Patient 2	0.67		1.14	
Patient 3	2.73		0.17	
Patient 4	2.61			0.98
Patient 5	2.97		6.54	
Patient 6	0.64	4.07		0.16
Patient 7			1.25	2.71
Patient 8	2.83		1.20	0.34
Patient 9	0.26		0.10	0.11
Patient 10			0.73	0.15
Patient 11			0.08	0.05
Patient 12			0.46	0.14
